# The assessment of inflammatory and structural lesions of the Achilles tendon by ultrasound imaging in inflammatory arthritis: a systematic review and meta-analysis

**DOI:** 10.1186/1757-1146-8-S2-P3

**Published:** 2015-09-22

**Authors:** Matthew Carroll, Nicola Dalbeth, Mark Boocock, Keith Rome

**Affiliations:** 1 Department of Podiatry, Auckland University of Technology, Health & Rehabilitation Research Institute, Auckland, 0627, New Zealand; 2 Department of Medicine, Auckland University, Auckland, New Zealand; 3 Department of Physiotherapy, Auckland University of Technology, Health & Rehabilitation Research Institute, Auckland, 0627, New Zealand

**Keywords:** Ultrasound, Achilles tendon, spondyloarthropathy, rheumatoid arthritis

## Background

Ultrasound (US) is a highly sensitive, reliable and non-invasive tool which allows for the assessment of inflammatory and structural lesions of tendons and entheseal sites. The aim of the systematic review and meta-analysis was to examine inflammatory and structural US lesions of the Achilles tendon (AT) in people with inflammatory arthritis compared to controls.

## Methods

An electronic literature search was performed on Medline, CINAHL, SportDiscus and The Cochrane Library. Methodological quality was assessed using a modified Quality Index. Odds ratios with 95% confidence intervals (CI) were determined. Meta-analysis was conducted on those studies considered to be homogenous.

## Results

Thirteen high to medium quality studies met the inclusion criteria. The majority of studies reported on US lesions in spondyloarthropathy (SpA), with limited evidence for other forms of IA. US lesions were not consistently defined with regard to Outcome Measures in Rheumatology Clinical Trials (OMERACT) definitions and numerous scoring systems were used across the majority of studies. The mean AT thickness at the enthesis in people with SpA was 0.54 mm thicker (95% CI 0.10 to 0.97 mm) with more frequent erosions in people with SpA (odds ratio (95%CI)) (7.43 (1.99 - 27.77), P = 0.003) and rheumatoid arthritis (RA) (odds ratio (95%CI) (9.60 (1.23 - 74.94), P = 0.03), compared to controls. There was no significant difference in the frequency of enthesophyte formation in people with SpA compared to controls (odds ratio (95%CI) (2.48 (0.64 - 9.70), P = 0.19).

## Conclusions

The systematic review identified that a majority of studies reporting US lesions indicative of inflammation and structural damage were in SpA, but limited evidence relating to other forms of inflammatory arthritis. Consistent application of the OMERACT US definitions and scoring of US lesions is required in future studies of AT disease in inflammatory arthritis.

